# The needs and experiences of mothers while in prison and post-release: a rapid review and thematic synthesis

**DOI:** 10.1186/s40352-021-00153-7

**Published:** 2021-11-12

**Authors:** Erica Breuer, Marc Remond, Stacey Lighton, Jane Passalaqua, Jennifer Galouzis, Kelly-Anne Stewart, Elizabeth Sullivan

**Affiliations:** 1 College of Health, Medicine and Wellbeing, University of Newcastle, Level 4 West, Hunter Medical Research Institute, Lot 1, Kookaburra Cct, New Lambton Heights, NSW 2305 Australia; 2 University of Technology, Sydney, Australia; 3 Corrective Services NSW, Sydney, Australia

**Keywords:** Mothers, Parents, Prison, Needs, Experiences, Review

## Abstract

**Background:**

Women in prison are a vulnerable group, often with a history of abuse, out-of-home care, mental health problems and unemployment. Many are mothers when they become involved in the criminal justice system and their gender and parenting related needs are often not considered. The aim of this rapid review was to thematically synthesize the existing research on the needs and experiences of mothers while in, and following release from, prison in Australia.

**Methods:**

We conducted a rapid systematic search of electronic databases, search engines, the websites of key agencies, and contacted key agencies and researchers.

**Results:**

Twenty-two publications from 12 studies met the inclusion criteria and were thematically synthesized in relation to the mothers, their children, family and community, and systems and services which mothers had contact with. We found that mothers in prison have a history of disadvantage which is perpetuated by the trauma of imprisonment. Release from prison is a particularly challenging time for mothers. In relation to their children, the included studies showed that the imprisonment of mothers impacts their maternal identity and role and disrupts the mother-child relationship. Specific strategies are needed to maintain the mother-child relationship, and to ensure the needs and rights of the child are met. In relation to family and community, we found that although family and social support is an important need of women in prison, such support may not be available. Moreover, the stigma associated with having been in prison is a significant barrier to transitions into the community, including finding employment and housing. In relation to systems and services, although limited services exist to support women in prison and on release, these often do not consider the parenting role. Evaluations of parenting programs in prison found them to be acceptable and beneficial to participants but barriers to access limit the number of women who can participate.

**Conclusion:**

Mothers have gender- and parenting-specific needs which should be considered in planning for corrective services in Australia. Any service redesign must place the woman and her children at the centre of the service.

## Background

Women in Australian prison constitute around 8% of the Australian prison population (Australian Bureau of Statistics, 2020) but the number of women in Australian prisons doubled between 2000 and 2018 (Word Prison Brief, 2018). Of these, approximately one third self-identify as Aboriginal or Torres Strait Islander (Australian Bureau of Statistics, 2019) which is substantially higher than the 3.3% of Aboriginal and Torres Strait Islander women in the Australian population (Australian Bureau of Statistics, 2018b).

About two thirds of women in prison self-identify as being a parent of at least one dependent child (Lobo & Howard, 2021). Upon entering prison, mothers with children under their care are at high risk of losing custody of their children either formally or informally (Dowell et al., 2018). Despite the rising numbers of women in prison, women have historically constituted a low proportion of the total prison population and therefore models of criminal justice, custody and post-release services have generally been developed around the needs of men (Baldry, 2010; Bartels et al., 2020).

Women in prison have a specific gendered set of needs and different pathways into prison (Stathopoulos & Quadara, 2014). They are a particularly marginalized group (Baldry, 2010), who often carry a history of childhood abuse (Stathopoulos & Quadara, 2014; Walker, 2018), exposure to violence (Day et al., 2018), complex mental health issues (Australian Institute of Health and Welfare, 2019; Butler et al., 2011) and substance abuse problems (Abbott, Magin, Davison, & Hu, 2017).

Compared to men, women in prison are more likely to: have experienced sexual violence (16.2% vs. 1.4%), have a history of depression (61.2% vs. 36.4%), self-harm (24.6% vs. 10.6%), report suicidal ideation (43.3% vs. 29.6%), and attempt suicide (28.6% vs. 17%)(Justice Health Forensic Mental Health Network, 2017; NSW Bureau of Crime and Statistics and Research, 2021). On average, the length of imprisonment for women in custody is shorter than for men in custody (221 vs. 430 days) (Justice Health Forensic Mental Health Network, 2017; NSW Bureau of Crime and Statistics and Research, 2021). Women also commit different types of offences. Acccording to the Australian Bureau of Statistics, in 2017 the most common serious offence for sentenced women was illicit drug offenses (21% of women in prison) whereas for men it was acts intended to cause injury for men (15% of men in prison). Women are more likely to be sentenced for fraud, deception and similar offences compared to men who were more likely to be sentenced for sexual assualt and related offences (Australian Bureau of Statistics, 2018a). Women have specific reproductive health needs in relation to pregnancy and childbirth (Bard et al., 2016) and have a high risk of returning to custody. A recent study in Queensland found that 70% women returned to custody within 2–4 years after release with 50% returning to custody within 1 year (De Rooy et al., 2019). Risk factors for re-offending included violent offenses, longer incarceration, younger age and identifying as Indigenous (De Rooy et al., 2019).

A similar proportion of men and women in prison are parents (Justice Health Forensic Mental Health Network, 2017). Imprisoned mothers and fathers face a variety of challenges such as separation from their children, maintaining their relationship while in prison, the recognition of their parenting role by the criminal justice system as well as ensuring care for their children when in prison (Bartlett et al., 2018; Flat Out Inc. and VACRO, 2006; Fowler et al., 2017; Walker, 2018). However, a greater proportion of women tend to have care responsibilities or sole custody of their children (Rossiter et al., 2016). This has important implications for the care of children during arrest, sentencing, imprisonment and release of mothers. In addition to the practical disruption to their lives, children of imprisoned parents may experience grief and loss, behavioural issues, stigma and discrimination and stress (Dawson et al., 2012). These needs and experiences are compounded in Aboriginal and Torres Strait Islander mothers who experience institutional racism and discrimination in prison (Kendall et al., 2020) in addition to intergenerational trauma and current and historical socio-economic disadvantage (Marmot, 2011; Shepherd et al., 2017). Aboriginal and Torres Strait Islander women are likely to be in prison as a result of minor offences, for example failing to pay a fine or public order offences, for repeated offenses (63% vs. 38% for women who do not identify as Aboriginal and Torres Strait Islander) and for shorter periods than women who do not identify as Aboriginal and Torres Strait Islander (17.7 months vs. 30.4 months) (Bartels, 2010).

Another key difference between women and men who are parents in prison is the extent to which women are informally held accountable for the impact of their imprisonment on their children (Elliot-Hohepa & Hungerford, 2013; Pösö et al., 2010; Walker, 2018). Women in residential programs for mothers and children in prison (‘prison nurseries’) describe being subjected to constant judgement of their parenting by custodial officers, other professionals in the prison environment, and other female prisoners (Shaw et al., 2015; Walker, 2018). Unintentionally, mothers and children’s residential programs become part of the architecture of power in prison environments as women are strongly incentivised to regulate their behaviour and try to repair their ‘spoiled’ maternal identity in order to gain a place in the program (Celinska & Siegel, 2010; Walker, 2018). Qualitative studies have found that these experiences can have health impacts including stress, anxiety, depression, feelings of hopelessness, and re-traumatisation, since only a minority of mothers in prison are offered a place in the programs (Celinska & Siegel, 2010; Walker, 2018).

The need for gender specific responses, including those that account for women’s mothering role, has been recognised, and strongly advocated for, over many years. This culminated in the ratification of the *United Nations rules for the treatment of women prisoners and non-custodial measures for women offenders* (the Bangkok Rules) (UN General Assembly, 2010). Internationally, best practices include changes to policies related to women entering into prison, for example suspended or alternative sentencing, providing accommodation which promotes health connections with family and friends, addressing both physical and mental health and socioeconomic determinants related to imprisonment, offering gender specific prison programs and transitional care, improving the prison environment and ensuring non-discriminatory security classification systems (Lorana Bartels & Gaffney, 2011). Examples include the Female Offender Intervention and Diversion Program in Oklahoma which provides community based supervision in conjunction with a holistic wraparound service and the Grand Valley Institution for Women in Ontario, Canada, which is a small women specific prison where babies can live with their mothers and range of services are available to women in prison and to prepare for release (Prison Reform Trust, 2013). In Australia, some progress has been made, though there is wide political, geographic, and demographic variation between the country’s eight States and Territories. Some have adopted an explicitly human-rights based approach to policy-making whilst others have adhered to a ‘tough on crime’ narrative, exhibiting rates of women’s imprisonment that are ‘beyond meaningful comparison’ (Baldry & Cunneen, 2014). The Better Pathways Framework in Victoria (Corrections Victoria, 2006) was a 4 year program aiming to reduce women’s imprisonment, increase access to services and reduce recidivism through 37 initiatives. Seven of the eight Australian jurisdictions allow younger children to live with their mothers in prison (Shlonsky et al., 2016). Although there are some targeted programs for pregnant women, mothers and children in prisons, including parenting programs (Rossiter et al., 2015, b; SHINE for Kids, 2020), there is wide variation between the Australian States and Territories (Walker, Baldry, & Sullivan, 2019). Programs can be difficult to access due to long waiting lists, lack of eligibility, especially for women on remand or short sentences, and lack of childcare in prison (Rossiter et al., 2016; Walker, 2018). Many are not designed for Aboriginal and Torres Strait Islander women (Council of Australian Governments, 2016). Again, On release from prison, access to transitional services remains limited: the Keeping Out of Women Coalition estimates that only 22% of women leaving prison in NSW receives services on release (Phelan, Sotiri, Scott, & Project, 2019) which include linkage to government funded healthcare services, government housing, employment and social services. This points to the need for systemic change to respond effectively to the needs to women in prison (Baldry, 2010; Bartels, Easteal, and Westgate, 2020; Bergseth, Jens, Bergeron-Vigesaa, & McDonald, 2011; Carlton & Segrave, 2015; Hannah-Moffat, 2001; Rossiter et al., 2015, b; Wright, Salisbury, & Van Voorhis, 2007).

While several studies have previously investigated the needs and experiences of mothers in prison in Australia (Bartels, Easteal, and Westgate, 2020; Burgess & Flynn, 2013; Minson, 2019; Walker, Baldry, and Sullivan, 2019), there has been no review which has systematically synthesized their needs and experiences, or the intersection of mother’s experiences with government services in Australia. We aimed to thematically synthesize existing research to understand the needs and experiences of mothers in prison while incarcerated and post-release in Australia in order to generate a knowledge-base that can be used to inform policy and practice in Australia. This includes understanding and challenges and difficulties faced by mothers in prison and on release. Although fathers in prison may have overlapping needs and experiences to mothers, especially in relation to their children, and mothers also have needs and experiences similar to women who are not parents, this study focuses on the literature at the intersection of gender and parenthood.

## Methods

We used a rapid review approach which maintains the rigour and transparency of a systematic review by outlining the search process, the criteria for inclusion of studies, the process of extraction of data, and the method of data analysis (Khangura, Konnyu, Cushman, Grimshaw, & Moher, 2012; Tricco et al., 2015). Prior to the review, the review team developed a protocol, which was approved by Corrective Services NSW.

### Search strategy

The review team developed a Population, Context, Phenomena of Interest, Study Design and Time Frame framework (Table 1) to identify search terms and specify the parameters used to guide the systematic search. We limited our review to Australia to ensure that our findings were specific to the needs and experiences of women in Australian prisons and the intersection between these and Australian services.

**Table 1:** Population, Context, Phenomena of Interest, Study Design and Time Frame

Criteria	Description	Search terms
**Population**	Mothers	Mother* Gestat* Parent* Matern* Wom?n* Child* Bab* Famil* Pregnan*
**Context**	In prison or those who have been released from prison in Australia	Prison* Custody Detention Crim* Correction* Incarcerat* Inmate Jail Offen* Imprison* Gaol Post-release Throughcare Reentry Limits: Australia English language
**Phenomena of interest**	Needs Experiences Experiences with the statutory systems Re-offending	Needs Hous* Health* Welfare Child protection Community Services Centrelink Out-of-home care Experience* Support Statutory system State Government Justice and Community Services Re-offending Recidivism Re-conviction Repeat offending Re-imprisonment
**Study design**	Qualitative and quantitative empirical studies	None
**Time frame**	Published from 2005 until end of August 2020	Limit: > 2004

We searched: 1) seven electronic databases (PubMed, Informit Humanities and Social Sciences, PsycInfo, ProQuest, EBSCOhost, Web of Science and Scopus); 2) search engines; and 3) websites of key agencies (including the Australian Institute of Criminology, the Australian Institute of Family Studies, the Analysis and Policy observatory and the Lowitja Institute). We also 4) emailed key researchers in the field, and the National Correctives Services Research Group, requesting any additional relevant research, including PhD theses. The search terms were modified to reflect specific requirements of each database that was searched and to improve the efficiency of the search in each database. This process was undertaken iteratively for each database searched.

Following the search, we exported the titles and abstracts of retrieved articles into Endnote (Clarivate Analytics, 2020) and removed duplicates. The remaining titles and abstracts were each screened by one member of the research team (EB, MR or SL) using pre-defined inclusion and exclusion criteria (Table 2). Each individual record was screened by one reviewer.

**Table 2:** Inclusion and Exclusion criteria

Inclusion criteria	
1.	Empirical research study using qualitative or quantitative research methods
**2.**	Includes research on the experiences, needs or risk of re-offending of mothers inprison or who were released from prison within 12 months of the date that the research was undertaken.
**3.**	Includes the experiences or needs of mothers and their children in contact with statutory or government systems while the women are in prison or within 12 months of release
**4.**	Research is conducted in Australia
**Exclusion criteria**	
**1.**	Systematic reviews (full text assessment was undertaken to check primary studies included in the rapid review)
**2.**	Commentary paper which does not provide research methods
**3.**	Not written in English
**4.**	Narrative reviews, conference abstracts and protocols
**5.**	Published prior to 2005
**6.**	Research on men
**7.**	Research on women who are not parents
**8.**	Full text not available

**Fig. 1:**
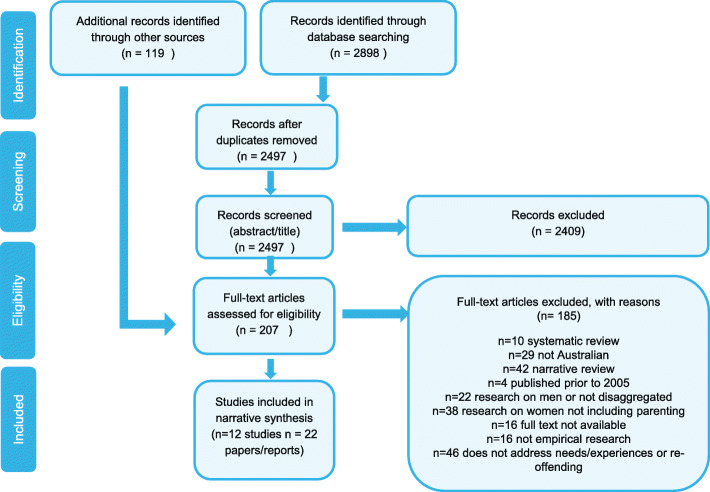
PRISMA Flow Diagram

After screening titles and abstracts, we obtained full-text versions of publications that met inclusion criteria. Each full text version was assessed by one reviewer (EB, MR or SL) for eligibility in discussion with research team. Any research reports obtained from grey literature databases, websites or through contacting experts were downloaded and the full text was screened for inclusion. This process is recorded in the PRISMA Flow Diagram (Fig. 1) (Moher, Liberati, Tetzlaff, & Altman, 2009).

### Data extraction

Data from each included full-text publication was extracted by one member of the research team (EB, MR or SL) using a data extraction form. The data extraction form included five critical appraisal prompts to assess the quality of the included publications (Dixon-Woods et al., 2006). However, we decided a priori not to exclude any publication based on methodological flaws. Due to the time constraints of this rapid review, we did not contact authors for additional information about published results.

### Data synthesis

To synthesize the data, we imported the full text of all included publications into NVIVO (QRS, 2011). We were guided by Thomas and Harden’s approach to thematic synthesis (Thomas & Harden, 2008):

1. A single reviewer for each research study coded the results and discussion sections of included publications line-by-line. A second reviewer reviewed the coding of a sample of studies and discussed this with the first reviewer. Papers were recoded where necessary to ensure a consistent approach across reviewers.
2. Identified codes were grouped into a hierarchical tree structure
3. The codes were used to identify descriptive and analytical themes. As this review focused on experiences both at the individual and system level, we adapted Bronfenbrenner’s ecological model of development (Bronfenbrenner, 1979) and structured the themes at individual, child, community and family, and systems and services levels.

We considered both the prison environment and the external environment, as well as the transitions between these. In the results section we include direct quotes from the primary studies. Where the quote is from an individual who was part of the primary study, we have indicated this in the text.

## Results

### Search results

As outlined in Fig. 1, database searches returned 2898 records. Following the removal of duplicates and title/abstract screening, 88 records were retained for full text screening. An additional 119 full-text publications were identified through other sources. A total of 22 publications encompassing 12 separate studies were included (see Table 3 for details). Ten (45%) of the publications were peer-reviewed publications and four (18%) were theses. Five of the 12 studies (6 of the 22 publications) were program evaluations relating to three programs.

**Table 3:** **Included studies and publications**

Study (Included publications)	Location	Aims	Study design (Methods of data collection)	Participants	Data analysis
**Descriptive Studies**					
Aboriginal Women with Dependent Children Leaving Prison Project (Baldry, 2009; Baldry etal., 2008a; Baldry etal., 2008b)	Western Sydney, New South Wales	To understand the needs of Aboriginal women exiting prison and their dependent children and the availability of services to address those needs	Qualitative study (interviews or focus groups with mothers and interviews with service providers)	*n* = 17 Aboriginal mothers < 6 months after exiting prison; Unknown number of agency and service providers	Qualitative analysis guided by the project logic
Mental illness as a mediator of mothers’ participation in the Victorian Criminal Justice System (Burgess, 2016)	Victoria	To examine the experiences of mothers with a mental illness, as they navigate Victoria’s criminal justice system, to plan the care of their children.	Exploratory embedded mixed methods approach (structured interview schedule)	*n* = 38 mothers in prison (*n* = 19 with and *n* = 19 without mental illness) *n* = 2 mothers released < 18 months ago (*n* = 1 with and *n* = 1 without mental illness); 4/38 mothers identified as Aboriginal or Torres Strait Islander	Quantitative data: descriptive Qualitative: thematic and content analysis
Children: Unintended victims of legal process – A review of policies and legislation affecting children with incarcerated parents (Flat Out Inc. and VACRO, 2006)	Victoria	To provide insight into the subjective effects of current Victorian laws and policies, regarding the children of women prisoners, on those who are enacting them, and those who are acted upon.	Qualitative study (qualitative interviews)	*n* = 15 mothers (12 in prison, 3 in community < 18 months post release), *n* = 12 police officers, *n* = 11 solicitors, *n* = 6 judges and magistrates, *n* = 12 interim carers of children of imprisoned women; 0/15 mothers identified as Aboriginal or Torres Strait Islander	Qualitative analysis (details not described)
The impact of maternal incarceration on adolescent children (Flynn, 2008, 2011, 2012; Flynn, 2013; Perry et al., 2011)	Victoria	To examine the impact of maternal imprisonment on 20 young people, aged between 10 and 18 years, whose mothers were incarcerated in the two women’s prisons in Victoria.	Qualitative study (in-depth semi-structured interviews)	Data from 20 adolescents was gathered from: n = 15 mothers (1–18 months post- release), *n* = 14 adolescent children (10–18 years); *n* = 3 professionals; 1/20 children reported Aboriginal or Torres Strait Islander heritage	Thematic analysis
I’m still your Mum: Mothering inside and outside prison (Stone, 2013) (Stone etal., 2015; Stone etal., 2017)	Victoria	To understand the effect which maternal incarceration has on the relationships between incarcerated mothers and their children.	Qualitative study (semi- structured in-depth interviews with professionals; discussions and meetings with key stakeholders)	*n* = 6 professionals who case-managed mothers while in prison and upon their release; meetings and discussions with *n* = 24 key stakeholders who advocate for incarcerated mothers. Aboriginal and Torres Strait Islander status of participants not reported.	Thematic analysis using a standpoint feminism perspective
Maternal incarceration, child protection, and infant mortality: a descriptive study of infant children of women prisoners in Western Australia (Dowell et al., 2018)	Western Australia	To describe the exposure of children aged less than 2 years to maternal imprisonment in Western Australia, their contact with child protection services, and infant mortality rate in Western Australia	Retrospective longitudinal cohort study using linked data	All children born in Western Australia between 1985 and 2011 whose biological mother was imprisoned at least once between their date of birth and 18th-birthday and a randomly sampled comparison group with no imprisonment match on age, gender and Aboriginal and Torres Strait Islander status.	Statistical analysis, using a log-binomial regression model
Women and Gestation in Prison: Becoming a ‘Good Enough Mother’(Walker et al., 2019; Walker, 2018)	7 prisons across the Australian Capital Territory, New South Wales, Northern Territory, Queensland, South Australia, Tasmania	To explain the institutional context for pregnancy and motherhood in prison and examine the “archaelogy” of current approaches	Qualitative (In depth interviews)	*n* = 25 imprisoned women (pregnant or had been pregnant in prison during the preceding 2 years); *n* = 14 correctional services staff; 7/25 women identified as Aboriginal or Torres Strait Islander	Social-structural Grounded Theory (Strauss & Corbin, 1997)
**Program evaluation**					
Sisters Inside Health Support Program Evaluation (ESSQ Community Services Consultancy, 2018)	Queensland	To evaluate the Sisters Inside throughcare post-release program focusing on women with dependent children	Participatory Action Research (interviews with support program workers, written feedback from program participants and monitoring data)	*n* = 6 Health Support Program workers; *n* = 13 program participants and program monitoring data from *n* = 109 participants; 4/6 Health Support workers and 73/109 program participants identified as Aboriginal and Torres Strait Islander	Not documented
Evaluation of the Parents Under Pressure program (Frye & Dawe, 2008)	Queensland	To determine whether women within the correctional system were prepared to engage with an intensive, individualised parenting program (PUP), and whether participation in such a program was associated with improved levels of maternal functioning and child behaviour.	Mixed methods pre post study (semi-structured interview and self-report questionnaires at baseline, post intervention and 3 months post intervention)	*n* = 12 mothers and their 12 children; Aboriginal and Torres Strait Islander status of participants not reported; 49/90 program participants identified as Aboriginal and Torres Strait Islander	ANOVA, comparison of scores to normative data.
Evaluation of Mothering at a Distance Evaluation 1 (Perry et al., 2009; Perry et al., 2011)	New South Wales	Evaluation of the development, implementation, effectiveness and sustainability of the Mothering at a Distance program	Mixed methods using appreciative inquiry approach (quantitative: participants, completion rates; data from the Department of Corrective Services’ Offender Integrated Management System (OIMS); qualitative: narrative-based interviews pre- and post-program and questionnaires at the completion of the group and 8 weeks; Facilitators: post- program interview and questionnaires)	*n* = 90 program participants; *n* = 30 facilitators; 49/90 program participants identified as Aboriginal and Torres Strait Islander	Quantitative: descriptive statistics; qualitative: content analysis using a priori themes
Evaluation of Mothering at a Distance Evaluation 2 (Rossiter et al., 2016)		To generate new knowledge about incarcerated parents and their parenting skills and knowledge, their learning and support needs, while in prison and when they return to the community. To evaluate two parenting programs, Mothering at a Distance (for Aboriginal mothers) and Hey Dad for Indigenous Dads, Uncles and Pops. (Only the evaluation of Mothering at a distance included in this review)	Mixed methods (survey of mothers in custody, interviews with stakeholders and review of evaluation data)	*n* = 64 mothers in prison; *n* = 19 key stakeholders; 52.3% of mothers identified as Aboriginal or Torres Strait Islander	Not documented
Evaluation of Mothering at a Distance Evaluation 3 (Rossiter et al., 2015, b)		To identify participants’ views on impact of the Mothering at a Distance program.	Mixed-methods study (open and closed questions in a survey for program participants)	*n* = 135 mothers; Aboriginal and Torres Strait Islander status of participants not reported.	Quantitative: descriptive statistics; Qualitative: in depth engagement and development of themes

**Fig. 2:**
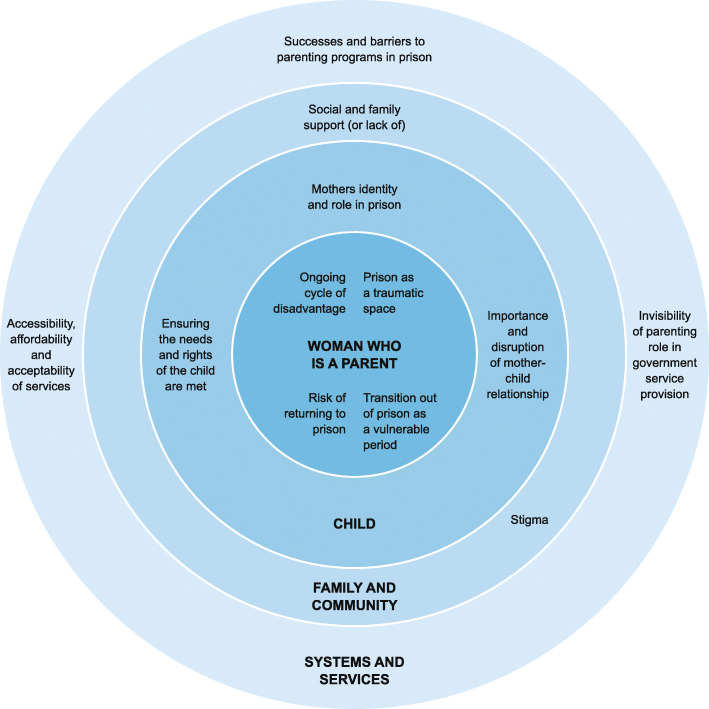
An overview of the themes related to the needs and experiences of mothers

Below we present the needs and experiences of mothers in prison and on release from the perspectives of the mothers, their children, professionals, and other stakeholders. The results are presented in relation to the individual woman/mother, the child, family and community, and systems and services. An overview of these results is provided in Fig. 2. The specific needs and the implications for systems and services are summarized in Table 4.

**Table 4:** Mothers’ needs as identified by women, stakeholders and professionals in relation to the identified themes in the review

Themes	Needs	In prison	Post-release	References
	**Individual Needs**			
**Prison as a traumatic space**	Culturally appropriate trauma informed support	x	x	(Baldry, 2009; Baldry et al., 2008a)
**Ongoing cycle of disadvantage**	Drug and alcohol services	x	x	(Burgess, 2016; ESSQ Community Services Consultancy, 2018; Stone, 2013)
	Mental health services	x	x	(Burgess, 2016; Stone, 2013; Walker et al., 2019)
	Domestic violence services	x	x	(Baldry, 2009; Baldry et al., 2008a)
	Family counselling	x	x	(Baldry, 2009; Baldry et al., 2008a; Frye & Dawe, 2008; Stone, 2013)
	Legal advice	x	x	(Baldry, 2009; Baldry et al., 2008a; Baldry et al., 2008b; Burgess, 2016)
	Physical health services	x	x	(ESSQ Community Services Consultancy, 2018)
	Transitional and long-term housing		x	(Baldry, 2009; Baldry et al., 2008a; ESSQ Community Services Consultancy, 2018; Flynn, 2008; Perry et al., 2009; Perry et al., 2011; Rossiter et al., 2015, b; Stone, 2013; Stone et al., 2015; Walker, 2018)
	Educational and vocational training	x	x	(Baldry et al., 2008a; Stone, 2013; Stone et al., 2015)
	Addressing poverty and other systemic factors which perpetuate crime		x	(Johnson et al., 2019; Stone, 2013)
**Transition out of prison as a vulnerable period**	Pre-release planning	x		(Flynn, 2008)
	Continuity of care		x	(Baldry, 2009; Baldry et al., 2008a; ESSQ Community Services Consultancy, 2018; Frye & Dawe, 2008; Stone, 2013; Stone et al., 2015; Stone et al., 2017)
	Services which address intersecting needs		x	(Burgess, 2016; ESSQ Community Services Consultancy, 2018; Frye & Dawe, 2008; Stone, 2013)
	Intensive post-release health support for women with complex health needs		x	(Burgess, 2016; ESSQ Community Services Consultancy, 2018; Frye & Dawe, 2008; Stone, 2013)
	**Needs related to children**			
**Mothers Identity and maternal role in prison**	Support for parenting and parenting skills	x	x	(Baldry, 2009; Baldry et al., 2008a; Baldry et al., 2008b; Flynn, 2008, 2014; Frye & Dawe, 2008; Perry et al., 2011; Rossiter et al., 2016; Rossiter et al., 2015, b; Stone, 2013; Walker et al., 2019; Walker, 2018)
	Involvement in planning care arrangements for children including time to plan and realistic expectations of risk of imprisonment at sentencing	x		(Burgess, 2016; Flat Out Inc. and VACRO, 2006; Flynn, 2008; Flynn, 2013)
	Access to parenting programs	x		(Frye & Dawe, 2008; Perry et al., 2009; Perry et al., 2011; Rossiter et al., 2016; Rossiter et al., 2015, b)
	Pre-release planning	x		(Flynn, 2008)
	Legal and other support to assist with reunification with children		x	(Burgess, 2016; ESSQ Community Services Consultancy, 2018; Flat Out Inc. and VACRO, 2006; Flynn, 2008, 2011; Frye & Dawe, 2008; Perry et al., 2009; Perry et al., 2011; Rossiter et al., 2016; Rossiter et al., 2015, b; Stone et al., 2015; Stone et al., 2017; Walker, 2018)
**Importance and disruption of mother-child relationship**	A range of options for positive ongoing contact, including predictable and high quality in-person visits with children in safe, conducive environments and affordable, flexible contact via phone and mail	x		(Baldry, 2009; Baldry et al., 2008a; Flat Out Inc. and VACRO, 2006; Flynn, 2008; Flynn, 2013; Flynn, 2014; Perry et al., 2009; Perry et al., 2011; Stone, 2013; Stone et al., 2017)
	Broader access to in-prison residential care arrangements for children that does not routinely exclude women on remand	x		(Walker, 2018)
	In-prison residential care arrangements for children	x		(Walker, 2018)
	Facilitation of contact through coordination with child protection services			(Flat Out Inc. and VACRO, 2006)
**Ensuring the needs and rights of the child are met**	**Children’s needs**			
	Housing	x	x	(Baldry, 2009; Baldry et al., 2008b; Burgess, 2016; Flat Out Inc. and VACRO, 2006; Perry et al., 2009; Una Stone et al., 2015; Stone et al., 2017)
	Care arrangements	x	x	(Burgess, 2016; Flat Out Inc. and VACRO, 2006; Flynn, 2008, 2011, 2012; Flynn, 2013; Stone, 2013; Stone et al., 2017; Walker et al., 2019)
	Schooling	x	x	(Baldry et al., 2008b; Flat Out Inc. and VACRO, 2006)
	Physical health	x	x	(Walker, 2018)
	Safety	x	x	(Flat Out Inc. and VACRO, 2006; Perry et al., 2009)
	Emotional support	x	x	(Baldry, 2009; Baldry et al., 2008a; Baldry et al., 2008b; Flat Out Inc. and VACRO, 2006; Flynn, 2008, 2014; Frye & Dawe, 2008)
	Contact with parent	x	x	(Baldry, 2009; Baldry et al., 2008a; Baldry et al., 2008b; Flat Out Inc. and VACRO, 2006; Flynn, 2008, 2014; Frye & Dawe, 2008).
	**Children who live in adult prisons**			
	Formalised support for mothers in mother and children units	x		(Walker, 2018)
	Access to healthcare	x		(Walker, 2018)
	Access to education	x		(Walker, 2018)
	**Needs related to community and family**			
**Social and family support (or lack thereof)**	Family and social support	x	x	(Baldry, 2009; Baldry et al., 2008a; Flat Out Inc. and VACRO, 2006; Flynn, 2008, 2011, 2012; Frye & Dawe, 2008; Perry et al., 2009; Rossiter et al., 2015, b; Stone, 2013; Walker, 2018)
**Stigma**	Non-stigmatising support services	x	x	(Rossiter et al., 2015, b)
	**Systems and services**			
**Accessibility, affordability and acceptability of services, including government and statutory services**	Comprehensive health services	x	x	(ESSQ Community Services Consultancy, 2018)
	Strengths based	x	x	(Rossiter et al., 2016; Stone, 2013)
	Provided by trusted individuals	x	x	(Baldry, 2009; Baldry et al., 2008b; ESSQ Community Services Consultancy, 2018; Frye & Dawe, 2008; Stone, 2013; Stone et al., 2015)
	Flexible	x	x	(Baldry et al., 2008a; Stone, 2013; Stone et al., 2017)
	Long term	x	x	(Frye & Dawe, 2008; Stone, 2013)
	Continuity between prison and release	x	x	(Baldry, 2009; Baldry et al., 2008a; ESSQ Community Services Consultancy, 2018; Frye & Dawe, 2008; Stone, 2013; Stone et al., 2015; Stone et al., 2017)
	Focus on rehabilitation	x	x	(Stone et al., 2015; Stone et al., 2017)
	Individualised and intensive	x	x	(Baldry, 2009; Baldry et al., 2008b; ESSQ Community Services Consultancy, 2018; Frye & Dawe, 2008; Stone, 2013; Stone et al., 2017)
	Co-ordinated across agencies	x	x	(Baldry, 2009; Baldry et al., 2008a; Baldry et al., 2008b; ESSQ Community Services Consultancy, 2018; Flat Out Inc. and VACRO, 2006; Flynn, 2008; Frye & Dawe, 2008; Stone, 2013; Stone et al., 2017)
	Culturally appropriate	x	x	(Baldry, 2009; Baldry et al., 2008a; Baldry et al., 2008b)
	Family focused child protection and housing services	x	x	(Stone et al., 2015)
**Invisibility of parenting role in government service provision**	Formal processes which recognise parenting status at all stages of contact with the justice system, including arrest, sentencing, imprisonment and release	x	x	(Flat Out Inc. and VACRO, 2006; Flynn, 2008)
	Formal supportive frameworks for residential mother’s and children’s programmes	x	x	(Walker, 2018)
**Successes and barriers to parenting programs in prison**	Access to parenting programs which address parenting issues for both younger and older children and acknowledge separation from children	x		(Perry et al., 2009; Perry et al., 2011)

### Needs and experiences related to the individual

#### In prison

##### Prison exacerbates an ongoing cycle of disadvantage

Mothers in prison have complex and intersecting needs many of which stem from social disadvantage and marginalization, poverty, early childhood abuse, sexual abuse, interpersonal violence, experiences of foster care, poor mental health, drug and alcohol abuse, low literacy and poor physical (Baldry, 2009; Baldry, J. Ruddock, & A. Taylor, 2008a; Burgess, 2016; Dowell, Mejia, Preen, & Segal, 2018; ESSQ Community Services Consultancy, 2018; Flat Out Inc. and VACRO, 2006; Flynn, 2008; Frye & Dawe, 2008; Stone, 2013; Stone, Liddell, & Martinovic, 2015; Stone, Liddell, & Martinovic, 2017; Walker et al., 2019; Walker, 2018). These needs include services related to drug and alcohol, mental health, domestic violence services, family counselling, legal advice, physical health in prison and post release and transitional and long term housing and addressing poverty and other factors which perpetuate crime (Baldry, 2009; Baldry et al., 2008a; Baldry, J. Ruddock, & J. Taylor, 2008b; Burgess, 2016; ESSQ Community Services Consultancy, 2018; Flynn, 2008; Frye & Dawe, 2008; Johnson et al., 2019; V. Perry, Fowler, & Heggie, 2009; Perry, Fowler, Heggie, & Barbara, 2011; Rossiter et al., 2015, b; Stone, 2013; Stone et al., 2015; Walker et al., 2019; Walker, 2018).

Prison exacerbates this cycle of disadvantage by separating mothers from their pre-prison life, including their jobs, homes and family. Women leaving prison contend with ongoing cycles of disadvantage. According to professionals working with mothers in prison, “*in most cases the mother is incarcerated for crimes underpinned by poverty, but the poverty does not disappear whilst she is in prison*” (Stone, 2013).

##### Prison is a traumatic space

Prison is, by design, a punitive environment which separates prisoners from their children, their family and the community. Mothers report “*bullying”*, threats of violence, and fear of other prisoners (J. R. Walker, 2018).. For example, one pregnant woman reported, “*… they were all having a go at me. And saying things to me like, “Whether you’re pregnant or not, you’re still gonna cop it”.”*

Separation from their children is a major stressor (Frye & Dawe, 2008; Stone, 2013; Stone et al., 2017), and according to professionals “*for most mothers … is the worst aspect of incarceration* (Stone, 2013). This separation disrupts the mother-child relationship children (ESSQ Community Services Consultancy, 2018; Rossiter et al., 2016; Rossiter et al., 2015, b; Stone, 2013; Walker, 2018) and as a result, mothers feel hopelessness and frustration (Baldry, 2009; Flynn, 2014; Walker, 2018) leading mothers to feel hopelessness and frustration (Baldry, 2009; Walker, 2018) (Flynn, 2014). While visitation from children and family may alleviate some of the pain of separation, mothers are frequently subjected to other forms of trauma, including strip searches, before obtaining visitation rights (Baldry, 2009; Flynn, 2014; Walker, 2018). Even where mothers live with their children in Mother and Children units, they often experience fear of imminent removal of their children and this fear is compounded by the strain of feeling that their mothering is surveilled (Walker, 2018). In addition to the needs described above, mothers need culturally appropriate and trauma informed support (Baldry, 2009; Baldry et al., 2008a).

Despite the trauma described above, for some mothers the institutional nature of the prison environment can represent a *“place of safety, security, routine, and regular meals*” in contrast to the “*chaos*” of life before prison (J. R. Walker, 2018).

#### After release

##### Transition out of prison is a vulnerable period

The transition out of prison is a vulnerable period for mothers. Upon release, mothers must organize housing, often in an environment where housing is difficult to obtain, meet statutory obligations, find employment or re-establish income support, as well as care for children or make progress towards reuniting with their children (Baldry, 2009; Baldry et al., 2008a; Baldry et al., 2008b; Burgess, 2016; ESSQ Community Services Consultancy, 2018; Flat Out Inc. and VACRO, 2006; Flynn, 2008, 2011; Perry et al., 2009; Perry et al., 2011; Rossiter et al., 2015, b; Stone, 2013; Stone et al., 2015; Walker, 2018). These responsibilities come abruptly after a period in prison during which they have had their daily basic needs taken care of (Stone et al., 2015) and where they have experienced a loss of agency (Flat Out Inc. and VACRO, 2006; Stone et al., 2015) and powerlessness (Walker, 2018). These challenges are often compounded by a background of ongoing disadvantage as described above.

Mothers find it difficult to meet post-release obligations and requirements, particularly if they receive no support or there is no co-ordination between agencies providing support (Baldry, 2009; Flat Out Inc. and VACRO, 2006; Stone, 2013). This results in mothers becoming overwhelmed (ESSQ Community Services Consultancy, 2018; Stone, 2013; Stone et al., 2015; Stone et al., 2017) and socially isolated, particularly where they are *“required to stay away from their ex-partner or former social circle.”*(Stone et al., 2015). Mothers exiting prison often have unstable housing arrangements and are at high risk of homelessness (ESSQ Community Services Consultancy, 2018; Flat Out Inc. and VACRO, 2006; Stone, 2013).

To meet these challenges mother need access to pre-release planning and continuity of care to services which address these intersecting needs, for example, intensive post-release health and other support (Baldry, 2009; Baldry et al., 2008a; Burgess, 2016; ESSQ Community Services Consultancy, 2018; Frye & Dawe, 2008; Stone, 2013; Stone et al., 2015; Stone et al., 2017).

### Needs and experiences related to the child

#### In prison

##### Maintaining mothers identity and maternal role in prison

The included studies show that mothers in prison struggle with their identity and role as mothers (Flat Out Inc. and VACRO, 2006; Flynn, 2008; Rossiter et al., 2016; Stone, 2013; Walker, 2018). Although parental roles may be taken into account during sentencing, the reality is that *“when mothers are imprisoned, these responsibilities are no longer seen to be competing; they are no longer mothers, just prisoners”* (Flynn, 2008). Furthermore, these mothers are often labelled as ‘*bad mothers’* by prison staff which is, at times, compounded by the mother’s own sense of guilt and shame related to being in prison (ESSQ Community Services Consultancy, 2018; Flynn, 2008; Frye & Dawe, 2008; Perry et al., 2009; Stone, 2013; Walker, 2018).

Maintaining a parental role and responsibility in prison is complicated by the physical separation from children (Flynn, 2008). Furthermore, even in Mother and Children Units, where young children can live with their mothers full-time, mothers report not being able to enact parenting decisions, including timely access to medical care for their child (Walker, 2018).

Mothers and other stakeholders identified the following needs to maintain mothers identity in prison: support for parenting and parenting skills and parenting programs, involving mothers in planning care arrangements for children including time to plan and realistic expectations of risk of imprisonment at sentencing, pre-release planning and providing access to legal and other support to assist with reunification with children (Baldry, 2009; Baldry et al., 2008a; Baldry et al., 2008b; Burgess, 2016; ESSQ Community Services Consultancy, 2018; Flat Out Inc. and VACRO, 2006; Flynn, 2008, 2011; Flynn, 2013; Flynn, 2014; Frye & Dawe, 2008; Perry et al., 2009; Perry et al., 2011; Rossiter et al., 2016; Rossiter et al., 2015, b; Stone, 2013; Stone et al., 2015; Stone et al., 2017; Walker et al., 2019; Walker, 2018).

##### Recognizing the importance and disruption of mother-child relationship in prison

Several studies reported that mothers in prison and other stakeholders identify children, and the maintenance of an ongoing mother-child relationship, as extremely important in the lives of these women (Baldry, 2009; Baldry et al., 2008a; Baldry et al., 2008b; Perry et al., 2009; Perry et al., 2011; Walker et al., 2019; Walker, 2018). Mothers worried about their children and their safety, “*and not being there for the children*” (Baldry, 2009; Baldry et al., 2008a; Burgess, 2016; Flat Out Inc. and VACRO, 2006; Flynn, 2008, 2012, 2014; Frye & Dawe, 2008; Perry et al., 2009; Walker et al., 2019). They were often unable to contribute to planning care arrangements for their children or other parenting decisions (Baldry, 2009; Burgess, 2016; Flat Out Inc. and VACRO, 2006; Flynn, 2012; Flynn, 2013; Flynn, 2014; Stone et al., 2017).

Predictable visits and phone calls can assist with maintaining the mother-child relationship despite physical separation (Flynn, 2008). However, the included studies report numerous barriers to visiting including: movement of women between prisons (Rossiter et al., 2016), unexpected lack of access to facilities (Rossiter et al., 2016), lack of carer support (including from welfare agencies), lack of disclosure to children about their mothers’ incarceration (Stone et al., 2017), the emotional toll of the visits on the mother and child (Perry et al., 2009), and prison environments which are unsuitable for children (Burgess, 2016; Flynn, 2014). These factors vary according to the prison risk classification (Flynn, 2014). Studies also highlighted barriers to phone contact including women only being permitted to call at specified times of day which may not work with the child’s schedule, the cost of the calls, and the willingness of the child’s carer to allow and facilitate contact (Flat Out Inc. and VACRO, 2006).

In order to maintain the mother-child relationship, mothers and stakeholders identified the need for a range of options for positive ongoing contact, including predictable and high quality in-person visits with children in safe, conducive environments and affordable, flexible contact via phone and mail, in-prison residential care arrangements for children which do not routinely exclude women on remand and facilitation of contact through coordination with child protection services (Baldry, 2009; Baldry et al., 2008a; Flat Out Inc. and VACRO, 2006; Flynn, 2008; Flynn, 2013; Flynn, 2014; Perry et al., 2009; Perry et al., 2011; Stone, 2013; Stone et al., 2017; Walker, 2018).

##### Ensuring the needs and rights of the child are met in prison and on-release

Imprisonment of mothers and the subsequent nature of the child (ren)‘s care arrangements greatly restrict women’s ability to act and make decisions in relation to the needs of their children. These needs include housing and care arrangements, schooling, physical health, safety, emotional support and contact with their parents (Baldry, 2009; Baldry et al., 2008a; Baldry et al., 2008b; Burgess, 2016; Flat Out Inc. and VACRO, 2006; Flynn, 2008, 2011, 2012; Flynn, 2013; Flynn, 2014; Frye & Dawe, 2008; Perry et al., 2009; Stone, 2013; Stone et al., 2015; Stone et al., 2017; Walker et al., 2019; Walker, 2018).

Care arrangements for children while the mother is in prison fall into three categories: 1) Mother and Children Units where children are housed in prison with their mothers; 2) kinship or informal care where children are cared for by family members or others; and 3) placement in out-of-home care. Each of these has specific challenges. In Mother and Children Units, mothers report being watched and judged by prison staff, receiving no assistance with child raising and care (except for informal arrangements with other prisoners to provide respite care), and trying to raise children when “*stripped of responsibility and autonomy.*” (Walker et al., 2019). Further challenges include the lack of services for children in prison, such as pre-school and healthcare (Walker, 2018). Kinship care, although common (Burgess, 2016; Flynn, 2008, 2012; Rossiter et al., 2016), is not always safe for children. For example, Walker reported that several women in her study had relied on abusive parents and ex-partners to care for their children (Walker, 2018). Out-of-home care is seen as a last resort by mothers because of concerns of rupturing the mother-children relationship and fear of abuse and neglect (Walker, 2018). These fears are often based on mothers’ own experiences of foster care. Despite this, Dowell and colleagues (2018) found that maternal incarceration results in a 27-fold increase in risk of out-of-home care placement for Indigenous children less than 2 years of age (RR 27.30; 95%CI 19.19 to 38.84, *p* < .001) and 110-fold for non-Indigenous Australian children (RR 110.10; 95%CI 61.70 to 196.49, *p* < .001).

### Needs related to community and family

#### In prison and after release

##### Social and family support (or lack thereof)

The studies included in this review highlight the need for family and other social support in providing care for children during their mother’s imprisonment and on her release (Baldry, 2009; Baldry et al., 2008a; Flat Out Inc. and VACRO, 2006; Flynn, 2008, 2011, 2012; Frye & Dawe, 2008; Perry et al., 2009; Rossiter et al., 2015, b; Stone, 2013; Walker, 2018). For example, mothers living with their child in the Mother and Children Unit are required to nominate an external carer for that child (Walker, 2018). However, many women in prison lack family support which has implications for both incarceration and the post-release phase (Stone, 2013). Even if women have a potentially supportive family, they may be required to stay away from what authorities regard as “negative” relations, leading to isolation and loneliness (Baldry, 2009).

##### Stigma

Mothers in, and following release from, prison are often stigmatized by prison staff, other government employees and the community (Rossiter et al., 2015, b; Stone, 2013; Stone et al., 2015; Walker, 2018). This stigma relating to having spent time in prison is often compounded by additional stigma arising from socio-economic disadvantage or from being Aboriginal (Walker, 2018). Such stigma is long lasting and impactful. It can result in women and their families avoiding contact with necessary health and social services (Rossiter et al., 2015, b) and affects women’s chances of obtaining housing, employment and reuniting with their children (Stone, 2013).

### Needs and experiences related to systems and services

#### In prison and after release

##### Accessibility, affordability and acceptability of services, including government and statutory services

In prison, mothers need access to comprehensive health services (ESSQ Community Services Consultancy, 2018). Mothers face several challenges with respect to the availability, accessibility and acceptability of, mental health, substance use and parenting programs in prison. These include the ad hoc availability of programs, lack of funding for external providers to provide programs, lack of knowledge about the availability of services for mothers, confusion about eligibility criteria, and lack of access to prisons for external service providers (Burgess, 2016; Flat Out Inc. and VACRO, 2006; Stone, 2013; Stone et al., 2017; Walker, 2018).

After release, the included studies highlight that women need comprehensive services to support their release, for example healthcare, transitional and long-term housing, employment and social services and transitional programs. However, these are limited (particularly in rural and remote areas), not culturally safe for Aboriginal women, and not coordinated with other services (Baldry, 2009; Baldry et al., 2008a; Baldry et al., 2008b; Burgess, 2016; ESSQ Community Services Consultancy, 2018; Flat Out Inc. and VACRO, 2006; Flynn, 2008, 2014; Frye & Dawe, 2008; Rossiter et al., 2016; Stone, 2013; Stone et al., 2017). One study found that staff of service agencies were inexperienced, utilised a deficit approach, had limited understanding of the difficulties mothers face when reintegrating into society, and had high levels of discrimination against the women (Stone, 2013; Stone et al., 2015; Stone et al., 2017).

The studies reflect the high level of interaction that women have with government agencies before, during and after they are imprisoned and the specific challenges the women face in regards to these interactions:

1. **Child protection services:** women and other stakeholders report the following challenges in interactions with child protection services: the removal of children in domestic violence situations where the mother is not the perpetrator; lack of communication with parents about taking the child into state custody or whose custody the children were in; constantly changing custody requirements; and not facilitating access to mothers for visits (Baldry et al., 2008a; Rossiter et al., 2016; Stone, 2013; Stone et al., 2015; Stone et al., 2017).
2. **Healthcare services:** mothers in prison have both negative and positive experiences with health services within prison and on release. One study reported that support workers expressed serious concerns about prison health services including: lack of thorough health checks on entry, lack of continuity of medication (including Opioid Substitution Therapy) on entry into prison, prescription of outdated medications, lack of access to the Pharmaceutical Benefits Scheme and Medicare coverage for prisoners, and the power prison officers exercise in preventing access to healthcare (ESSQ Community Services Consultancy, 2018). However, despite these challenges, a basic level of health services is available in Australian prisons (Stone, 2013). The most critical challenge identified was the lack of continuity between prison and post-release healthcare (ESSQ Community Services Consultancy, 2018; Stone et al., 2017).
3. **Public housing:** studies reveal that mothers exiting prison face substantial barriers when trying to access public housing. (Baldry, 2009; Baldry et al., 2008a; Baldry et al., 2008b; Flynn, 2011). These include: being classified as medium rather than high need for public housing, inappropriate location of housing (e.g., in a known drug hot spot), and the lack of suitable housing appropriate for reunification with children (Stone, 2013) .
4. **Legal services** are important for women to navigate both the criminal justice system and the family court. However, lack of knowledge was the key challenge related to legal services described in the included studies. This encompassed a lack knowledge about the legal system (Burgess, 2016), poor communication from the assigned legal team (Flat Out Inc. and VACRO, 2006), and a lack of information about the availability of Legal Aid (Baldry et al., 2008a).

##### Invisibility of parenting role in service provision

Many of the included studies highlight the lack of a parenting inclusive approach to services and a lack of coordinated response to children’s needs (Baldry, 2009; Baldry et al., 2008a; Baldry et al., 2008b; Burgess, 2016; ESSQ Community Services Consultancy, 2018; Flat Out Inc. and VACRO, 2006; Flynn, 2008; Rossiter et al., 2016; Rossiter et al., 2015, b; Stone, 2013; Stone et al., 2015; Stone et al., 2017; Walker et al., 2019; Walker, 2018). These gaps are apparent at different points in the course of women’s contact with the justice system, including at the intersection of child welfare and adult legal systems (Flynn, 2008). For example, in Victoria, there is a lack of formal policies relating to primary caregivers and the care of children at arrest, during sentencing, and on release of their caregivers (Flat Out Inc. and VACRO, 2006). This results in difficulties for mothers in arranging secure temporary and long-term care arrangements for their children, planning for handover (Flynn, 2008; Flynn, 2013) of children, and, ultimately, family reunification (Flat Out Inc. and VACRO, 2006). Mothers and stakeholders identify the need for formal processes which recognise parenting status at all stages of contact with the justice system, including arrest, sentencing, imprisonment and release and supportive frameworks for residential mother’s and children’s programs (Flat Out Inc. and VACRO, 2006; Flynn, 2008) (Walker, 2018).

##### Successes and barriers to parenting programs in prison and on release

This review included evaluations of the in prison Mothering at a Distance (MAAD) program (Perry et al., 2009; Perry et al., 2011; Rossiter et al., 2016; Rossiter et al., 2015, b) and the Parenting Under Pressure (Frye & Dawe, 2008) transitional program. The evaluation of the Parenting Under Pressure program (Frye & Dawe, 2008), an intensive, individualised parenting program which aimed to improve maternal functioning and child behaviour, showed significant improvement in maternal functioning, parent-child functioning, and child behaviour from pre-treatment to post-treatment and follow-up. However, the sample size for this evaluation was small so that results must be treated cautiously. Evaluations of the MAAD program which centered around supporting mothers in their role, teaching mothers practical strategies with children, and mothering from prison, reported that participants were satisfied with the program and found it to be a positive experience (Perry et al., 2009; Rossiter et al., 2016; Rossiter et al., 2015, b). Barriers to participation in the MAAD program include women moving between correctional centres (Rossiter et al., 2016), not being able to participate while on remand or on short sentences (Rossiter et al., 2016), lack of appropriate facilities to conduct participant groups (Perry et al., 2009), distractions and delays due to the prison environment (Perry et al., 2009), and not being offered the program (Rossiter et al., 2016).

## Discussion

This review framed the needs and experiences of mothers in and leaving prison through the lens of the individual, child, community and family, and systems and services in Australia. The findings across the 12 studies are consistent with previous research about women in prison in Australia (Baldry, 2010; Hale) suggesting that prior to prison women often occupy marginalised spaces in society, with substantial health problems and other systemic difficulties (Baldry, 2010; Kendall, Lighton, Sherwood, Baldry, & Sullivan, 2019). The co-occurrence of mental health issues, substance abuse and trauma are common for incarcerated women (Hale; Hayes, 2015; Johnson, 2006; Lee, Harrison, Mills, & Conigrave, 2014). Women are often imprisoned for short periods of time and their previous trauma is likely to be exacerbated by imprisonment (Bartels et al., 2020; Hale). This is supported by qualitative work which reported that continuity of the mother-child relationship and, at times, repair and restoration of that relationship, is vital to the mental health and social and emotional wellbeing of mothers with incarceration experience. Repeated separation and disruptions between mother and child are implicated in trauma and cycles of repeated incarceration (Lighton, 2019).

Upon release, women return to marginalised spaces in society. Mothers receive insufficient support to reintegrate into the community (Baldry, 2010; Hale, 2020). This perspective is corroborated by the findings of studies which explored the post-prison transition period for women in Australia and reported that there was little continuity of healthcare between prison and community (Abbott, Magin, Lujic, & Hu, 2017) and that this is exacerbated by stigma and exclusion from care (Abbott, Magin, Lujic, et al., 2017). Because of the complex needs of many women in prison, they require services in prison and post-release which address intersecting needs such as drug and alcohol use, mental health, domestic violence, physical health, transitional and long-term housing, educational and vocational training and legal advice ((Baldry, 2009; Baldry et al., 2008a; Baldry et al., 2008b; Burgess, 2016; ESSQ Community Services Consultancy, 2018; Flynn, 2008; Frye & Dawe, 2008; Perry et al., 2009; Perry et al., 2011; Rossiter et al., 2015, b; Stone, 2013; Stone et al., 2015; Walker et al., 2019; Walker, 2018). These services should be culturally safe for Aboriginal women, trauma-informed and provide continuity of care while addressing poverty and other systemic factors which perpetuate crime (Baldry, 2009; Baldry et al., 2008a; ESSQ Community Services Consultancy, 2018; Frye & Dawe, 2008; Stone, 2013; Stone et al., 2015; Stone et al., 2017; Walker et al., 2019).

We found that mothers have additional support needs in relation to their children. Of primary importance is the need to maintain relationships with their children while in prison and to re-establish these relationships upon release (Baldry, 2009; Baldry et al., 2008a; Flat Out Inc. and VACRO, 2006; Flynn, 2008; Flynn, 2013; Flynn, 2014; Perry et al., 2009; Perry et al., 2011; Stone, 2013; Stone et al., 2017). This review, together with the broader research in Australia on the impact of incarceration on primary caregivers, demonstrates the importance of considering the needs and rights of children during periods when their parents interact with the criminal justice system. Although this review did not specifically include or synthesize data from the arrest and sentencing period, available Australian literature concerning arrest and the responsibilities of the parent and needs of the child, indicate that children are often suddenly left without adequate care arrangements (Flat Out Inc. and VACRO, 2006; Flynn, 2013). This review has identified the importance of meeting children’s basic needs but also revealed the need to ensure their emotional support and safety, and the importance of meeting the cultural needs of Aboriginal and Torres Strait Islander children (Baldry, 2009; Baldry et al., 2008a; Baldry et al., 2008b; Flat Out Inc. and VACRO, 2006; Flynn, 2008, 2014; Frye & Dawe, 2008; Perry et al., 2009). This is in line with growing international evidence that child responsive caregiving, physical health, adequate nutrition, opportunities for early learning, and security and safety allow children to develop into healthy resilient adults (Richter et al., 2017).

A key finding in the review which is reflected in the wider Australian literature is the lack of co-ordination between government and non-government services and agencies, and the lack of continuity of services in prison and post-release (Abbott, Magin, Davison, et al., 2017; Abbott, Magin, Lujic, et al., 2017; Carlton & Segrave, 2015; Hale, 2020; McIvor et al., 2009; Trotter et al., 2012). It is problematic that women exiting prison are required to negotiate access to multiple social, family and health services. In addition, we found that the systems did not explicitly support the parenting role; this finding is particularly relevant to child protection systems and the criminal justice system (Flat Out Inc. and VACRO, 2006; Flynn, 2008) which must work more collaboratively to ensure that the needs of the family are prioritized (Trotter et al., 2015). This issue has been highlighted by a substantial body of research on parenting and the criminal justice system (Flynn, Bartlett, Fernandez Arias, Evans, & Burgess, 2015; Flynn, Naylor, & Arias, 2016; Fowler et al., 2017; He & Flynn, 2019; Pridmore et al., 2017; Saunders & MacArther, 2013; Sheehan, 2010; Trotter et al., 2015). Importantly, the agencies and services do not always consider the specific needs of Aboriginal and Torres Strait Islander women and their children including considering the effects of intergenerational trauma, child removal, and culturally safe models for services and programs (Baldry, 2009; Baldry et al., 2008a; Baldry et al., 2008b; Walker et al., 2019).

### Recommendations for practice

In light of these findings, we recommend a redesign of systems, policies and practice so that they centre on the needs of mothers in prison and promote reintegration into the community post-release. This includes looking critically at non-custodial options such as community-based orders and home detention. As part of an integrated approach to reducing the risk of a return to prison, any redesign should aim to minimize the trauma of imprisonment, recognize and address systemic disadvantage and complex individual needs of mothers in prison, and support women transitioning from prison into community. Ideally, mothers should be provided with integrated, flexible, long term services that provide continuity of care from initial contact with the criminal justice system until release, providing targeted support mothers. These services should include: domestic violence services (Baldry, 2009; Baldry et al., 2008a), family counselling (Baldry, 2009; Baldry et al., 2008a; Frye & Dawe, 2008; Stone, 2013), legal advice ((Baldry, 2009; Baldry et al., 2008a; Baldry et al., 2008b; Burgess, 2016), and transitional and long term housing (Baldry, 2009; Baldry et al., 2008a; ESSQ Community Services Consultancy, 2018; Flynn, 2008; Kendall et al., 2019; Perry et al., 2009; Perry et al., 2011; Rossiter et al., 2015, b; Stone, 2013; Stone et al., 2015). These could be provided through targeted individualised multidisciplinary services using a case work approach (Baldry, 2009).

In relation to children, policies should support the maternal role in prison and post-release, facilitate the maintenance of the mother-child relationship and ensure the needs and rights of the child are met. This could be achieved by using a child rights based framework to ensure that the rights of the child are met (UN General Assembly, 1989). Mothers in prison must be supported to meet the developmental needs of their children including responsive caregiving, physical health, adequate nutrition, opportunities for early learning, and security and safety (Richter et al., 2017). This applies whether the children are living with their mother in prison or where they have other care arrangements.

In relation to family and community, we recommend the services and policies are put in place to recognize that not all mothers can reliably access family support when in prison and on release (Baldry, 2009; Baldry et al., 2008a; Flat Out Inc. and the Victorian Association for the Care and Resettlement of Offenders (VACRO), 2006; Flynn, 2008, 2011, 2012; Frye & Dawe, 2008; Perry et al., 2009; Rossiter et al., 2015, b; Stone, 2013; Walker, 2018). In addition, it is important to consider interventions to reduce stigma, for example, acknowledging and conducting stigma reduction training for prison officers (Flat Out Inc. and VACRO, 2006) and supporting initiatives to address community stigma.

At the systems and services level, the focus needs to be on improving the accessibility, acceptability, and co-ordination of services; ensuring a parenting-inclusive approach in all government service agencies; and supporting comprehensive parenting services. One example of this approach would be formalising policies which ensure mother’s parental role, and the rights of the child are met at each point of contact with the criminal justice system, including during arrest and sentencing (Burgess, 2016; Flynn, Bartlett, Arias, Evans, & Burgess, 2015; Trotter, Flynn, & Baidawi, 2017; Trotter et al., 2015), and also when accessing other services such as child protection, housing, health and welfare.

### Limitations of the review

This review only included studies focused on mothers in prison and during the post-release period and therefore many of the studies had a parenting specific focus rather than the broader needs and experiences of mothers beyond their parenting role. Because of the small number of included studies which considered Aboriginal and Torres Strait Islander women in depth, we were not able to separately consider the needs and experiences of this population. Note, however, that this topic has been covered in detail elsewhere (Jones et al., 2018). Similarly, the focus of this review on mothers in prison did not allow for an in-depth analysis of the needs and experiences of the children with a mother in prison. Such a review is called for so that these issues can be elucidated and potential means to address them explored (Burgess & Flynn, 2013). We also recognize the role of fathers as parents and primary caregivers and their experience in and on release from prison is important and has been explored elsewhere (Bartlett, 2019; Bartlett et al., 2018; Dennison, Smallbone, Stewart, Freiberg, & Teague, 2014; Flynn, 2012; Fowler et al., 2017) and may benefit from a similar thematic synthesis to understand the similarities and differences between mothers and fathers in prison.

Our thematic synthesis highlighted the paucity of primary studies in this area. Our analysis is based on data from only 12 studies (22 publications) and therefore may not cover all the needs and experiences of Australian mothers in prison and on release. Further primary research is these areas is encouraged.

## Conclusion

The gender- and parenting-specific needs of mothers in and leaving prison should be considered a priority in planning for corrective services in Australia. This requires a wholistic review and redesign of corrective services, policies and systems to ensure that the needs of mothers are met in a variety of contexts: 1) as individuals who often have a history of disadvantage and trauma exacerbated by prison, 2) as parents who need to maintain and/or strengthen their relationships and parenting role with their children in order to ensure that the needs of their children are met; 3) as members of families and the broader community; and 4) as consumers of services that they engage with throughout their contact with the criminal justice system and on release.

## Data Availability

All data generated or analysed during this study are included in this published article.
